# Structural Evolution of Paramagnetic Lanthanide Compounds in Solution Compared to Time- and Ensemble-Average Structures

**DOI:** 10.1021/jacs.3c01342

**Published:** 2023-06-16

**Authors:** Barak Alnami, Jon G. C. Kragskow, Jakob K. Staab, Jonathan M. Skelton, Nicholas F. Chilton

**Affiliations:** Department of Chemistry, The University of Manchester, Manchester M13 9PL, U.K.

## Abstract

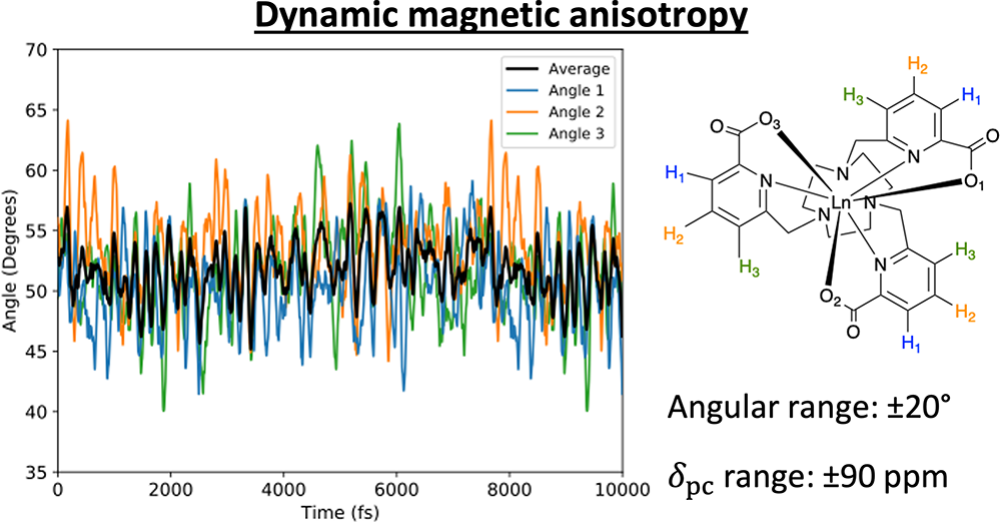

Anisotropy in the magnetic susceptibility strongly influences the paramagnetic shifts seen in nuclear magnetic resonance (NMR) and magnetic resonance imaging (MRI) experiments. A previous study on a series of C_3_-symmetric prototype MRI contrast agents showed that their magnetic anisotropy was highly sensitive to changes in molecular geometry and concluded that changes in the average angle between the lanthanide–oxygen (Ln–O) bonds and the molecular C_3_ axis due to solvent interactions had a significant impact on the magnetic anisotropy and, consequently, the paramagnetic shift. However, this study, like many others, was predicated on an idealized C_3_-symmetric structural model, which may not be representative of the dynamic structure in solution at the single-molecule level. Here, we address this by using ab initio molecular dynamics simulations to simulate how the molecular geometry, in particular the angles between the Ln–O bonds and the pseudo-C_3_ axis, evolves over time in the solution, mimicking typical experimental conditions. We observe large-amplitude oscillations in the O–Ln–C̃_3_ angles, and complete active space self-consistent field spin–orbit calculations show that this leads to similarly large oscillations in the pseudocontact (dipolar) paramagnetic NMR shifts. The time-averaged shifts show good agreement with experimental measurements, while the large fluctuations suggest that an idealized structure provides an incomplete description of the solution dynamics. Our observations have significant implications for modeling the electronic and nuclear relaxation times in this and other systems where the magnetic susceptibility is exquisitely sensitive to the molecular structure.

## Introduction

Magnetic resonance imaging (MRI) is a powerful diagnostic tool in medicine and biomedical research due to its ability to image soft tissue, and the development of contrast agents has substantially improved the quality of information that can be obtained from MRI imaging.^1^ In recent years, Gd(III) complexes have dominated the development of MRI contrast agents,^2^ due to the large magnetic moment and the lack of ground-state orbital degeneracy in the Gd(III) ion resulting in long electronic spin relaxation times (*T*_1_) and fast nuclear spin relaxation rates (*R*_1_) in its vicinity.^2,3^ While modulation of *R*_1_ is the dominant mechanism for obtaining contrast in MRI, alternative strategies exploiting the paramagnetic shifts of magnetically anisotropic ions (for example Dy(III)) are also a possibility. So-called PARASHIFT agents have been shown to return signals that are sensitive to local temperature and pH,^4^ allowing a single MRI scan to provide more information than just the location of the agent.^5^

The paramagnetic shift arises from the delocalization of spin density from the metal onto bonded atoms (the contact shift, δ_c_) and from the through-space magnetic dipolar contribution (the pseudocontact shift, δ_pc_). For the trivalent lanthanide ions Ln(III), whose 4f electron density is very well localized, δ_c_ is often negligible and the paramagnetic shift is dominated by δ_pc_. The solution-averaged pseudocontact shift arises from the magnetic anisotropy of the metal ion, which itself arises from the contribution of the orbital angular momentum (*L*) to the total angular momentum (*J*), and which is affected by the non-spherical environment of the molecular complex. The effects of the crystal field (CF) or ligand field (LF) on the orbital angular momentum are often unknown, and Bleaney’s simplified theory allows a connection between δ_pc_ and the CF (eq 1; note that herein we limit our discussion to the paramagnetic shifts of the ^1^H nuclei, but all NMR active nuclei will be subject to paramagnetic effects):^6−8^
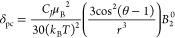
1Here, *B*_2_^0^ is the axial CF parameter, *C_J_* is a Bleaney constant, and θ and *r* are the spherical coordinates of a given proton relative to the symmetry axis of the molecule. However, one must note that the CF potential of a Ln(III) ion contains many more terms than *B*_2_^0^, and this model essentially folds all the CF information into one parameter. The *B*_2_^0^ value determined using Bleaney theory is therefore not the “true” *B*_2_^0^ one would obtain from spectroscopic measurements or ab initio theory. A common assumption is that an isostructural series of Ln(III) complexes should have similar CF parameters, which is a good approximation in some cases, and would lead to δ_pc_ being linearly related to *C_J_*.^9−11^However, this is not always the case due to subtle structural changes across the series that can lead to significant changes in *B*_2_^0^.^5^ Nonetheless, experimentally measured pseudocontact shifts provide rich information for determining the structure of metalloprotiens^12−19^ by allowing the position of the metal ion to be placed relative to the protons.

**Figure 1:**
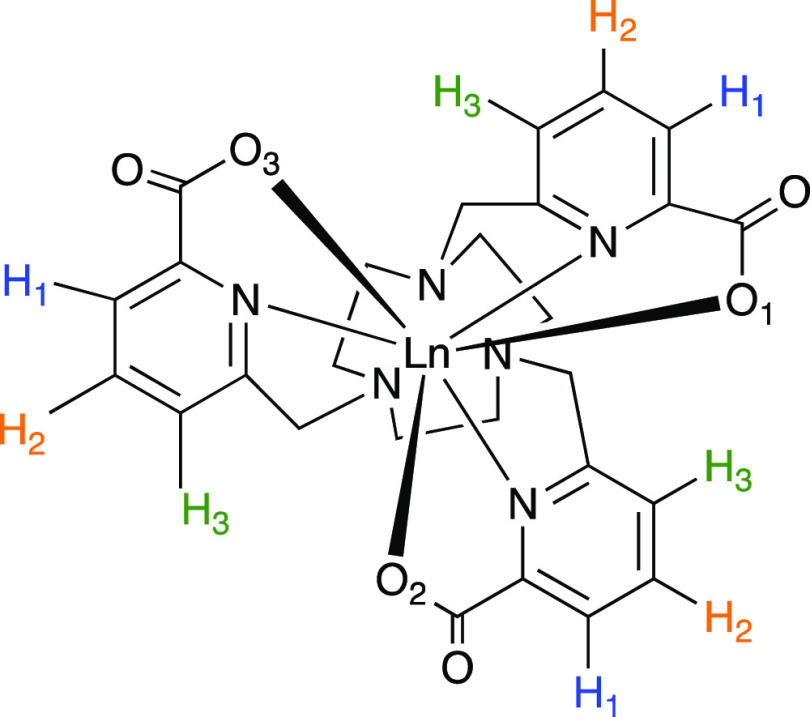
Chemical structure of [LnL^1^].^6^

Numerous groups have been investigating the connection between molecular structure, magnetic anisotropy, and pseudocontact shifts.^5,20−22^A recent study of a series of C_3_-symmetric complexes, [LnL^1^] (L^1^ = 1,4,7-tris[(6-carboxypyridin-2-yl)methyl]-1,4,7-triazacyclononane; Figure 1), led to the interesting observation that a number of different Ln(III) analogues had the same sign of δ_pc_ despite differing signs of *C_J_*, and that δ_pc_ had a significant solvent dependence.^6^ Even stronger solvent dependencies were later reported for related compounds.^23^ Neither of these effects can be explained using the simplified Bleaney theory. They arise due to the inherent near-zero *B*_2_^0^ in these compounds, which is a unique situation caused by the axial CF potential of the triazacyclononane N_ax_ atoms being of the same magnitude and opposite sign to the equatorial CF potential of the pyridyl N_eq_ atoms, and the axial carboxyl O atoms being positioned near the “magic angle” where their contribution to *B*_2_^0^ is zero. Hence, the overall sense of magnetic anisotropy in these compounds can be modulated by choice of Ln(III) and the solvent polarity, which both cause small structural changes that lead to changes in the CF and hence the magnetic anisotropy.^6^ Indeed, minimization of the magnetic anisotropy appears to correlate with the performance of Gd(III) compounds as dynamic nuclear polarization agents,^24^ and thus these results should be of interest to that community.

In our previous study, we performed an ab initio study of an optimized C_3_-symmetric structure to examine how the angle between the Ln–O bonds and the C_3_ axis affected the CF, which indicated that changes of a few degrees in these angles could change the sign of the magnetic anisotropy from positive to negative. Based on these results, we hypothesized that solvent polarity and hydrogen-bond affinity could perturb the average O–Gd–C_3_ angle, leading to the observed changes in δ_pc_. While sufficient to explain the experimental data, this approach assumed an average structural model, and we have no direct evidence linking the molecule–solvent interactions and experimental observations.

In this study, we generate a more realistic model of the solution structure of the [GdL^1^] complex in D_2_O, MeOD, and deutero-dimethyl sulfoxide (d_6_-DMSO) using ab initio molecular dynamics (AIMD) simulations based on density-functional theory (DFT). We use Gd(III) for these simulations as it is in the center of the lanthanide series and thus is a good structural approximation for the other lanthanides. The AIMD approach allows us to model the evolution of the structure over time in the presence of explicit solvent molecules, hence more accurately mimicking experiments than an average structural model. We observe solvent-induced changes in the time-averaged O–Gd–C̃_3_ angles, on the order of those observed experimentally, but with substantial oscillations and asymmetry at the single-molecule level. We subsequently study the effects of dynamic magnetic anisotropy in [DyL^1^] by substituting the Dy(III) ion in place of Gd(III) in the AIMD trajectories. We do this by performing complete active space self-consistent field spin–orbit (CASSCF-SO) calculations at snapshots during the MD trajectories to obtain the CF Hamiltonian and magnetic susceptibility tensor, and hence directly determine the δ_pc_ for [DyL^1^] in the three solvents. The large structural fluctuations and the sensitivity of the magnetic anisotropy to the structure lead to large fluctuations in δ_pc_, on the order of ±100 ppm compared to the experimental δ_pc_ on the order of 10 ppm. While these short-timescale AIMD simulations on a single molecule are insufficient to obtain time- and ensemble-averaged properties in perfect agreement with the experiment, the results from our AIMD trajectories are much closer to the experiment than using optimized or average structure models. Furthermore, the large oscillations in the structure revealed by these calculations, and hence the larger oscillations in magnetic anisotropy at the single-molecule level, will have a significant impact on the magnetic relaxation timescales, and thus on the design of novel PARASHIFT agents.

## Experimental Method

AIMD calculations were performed on a fully-deuterated Gd(III) analogue d-[GdL^1^], starting from a gas-phase optimized structure,^6^ using VASP 6.2.0.^25,26^ We used Gd(III) as it is in the middle of the lanthanide series and thus is a good structural model for other Ln(III) ions for this and subsequent studies.

To construct the solvated systems with MeOD and D_2_O, solvent molecules were initially placed on a regular grid and equilibrated using a 100 ps classical MD simulation at *T* = 300 K with Tinker v8.10.1^27^ and the OPLS-AA force fields^28^ with an *NVT* ensemble. A cavity was then created at the center of the box by removing an appropriate number of solvent molecules, into which the optimized d-[GdL^1^] molecule was placed. To construct the solvated system with d_6_-DMSO, for which the OPLS-AA force field is not defined, a model with d-[GdL^1^] at the center of the box was created without optimizing the initial solvent configuration and the combined molecule-solvent system equilibrated using a 10 ps AIMD simulation at 300 K. The three initial solvated models were then optimized via a two-stage process: in the first stage, we optimized the atomic positions with the box size (i.e., simulation cell volume) fixed, and in the second stage we froze the atomic positions and optimized the cell volume, while maintaining the cubic cell shape, to equilibrate the pressure to as close to zero as possible. This led to box sizes of (19.86 Å),^3^ (19.94 Å),^3^ and (19.56 Å)^3^ for the D_2_O, MeOD, and d_6_-DMSO models, respectively. Finally, we ran “production” AIMD simulations at 300 K for 10 ps with a timestep of 1 fs (i.e., 10,000 timesteps).

Electron exchange and correlation were described using the Perdew–Burke–Ernzerhof (PBE) generalized gradient approximation (GGA)^29^ in conjunction with the Grimme D3 dispersion correction (i.e., PBE-D3).^30^ The ion cores were modeled using the projector augmented-wave (PAW) method,^31,32^ using the 4f-in-core potential for Gd(III). For the box sizes of ∼(20 Å)^3^ used in the calculations, a plane-wave cut-off and *k*-point grid of 600 eV and 1 × 1 × 1 (i.e., Γ-point only) respectively, were used for representing the valence electronic structure. During the production MD calculations, the automatic machine learning force fields implemented in VASP were employed in order to increase the computational efficiency.^33−35^

Subsequently, a series of CASSCF-SO calculations were performed on the Dy(III) analogue [DyL^1^] with OpenMolcas 22.06,^36,37^ generated by extracting [GdL^1^] configurations from every 30^th^ frame of the AIMD trajectory (i.e., every 30 fs) and replacing the Gd atoms with Dy. The Dy atoms were treated with the ANO-RCC-VTZP basis set, the N and O donor atoms with the ANO-RCC-VDZP basis set, and all other atoms with the ANO-RCC-VDZ basis set.^38,39^ The two-electron integrals were decomposed using the Cholesky method with a high threshold of 10^–8^. The electronic configuration of Dy(III) (4f^9^) was modeled with a complete active space of nine electrons in the seven 4f orbitals, where we considered 18 roots of the *S* = 5/2 spin ground state. To mimic the solvation environment of [DyL^1^], one unit-cell’s worth of whole solvent molecules were individually treated as a set of point charges and dipoles obtained from a single-point DFT calculation (GAUSSIAN 09,^40^ PBE,^29^ cc-pVDZ basis set^41^) followed by a CHelpG charge decomposition of the electrostatic potential.^42^ From these CASSCF-SO calculations, we directly obtain the complete magnetic susceptibility (**χ**) tensor, which allows us to calculate the pseudocontact shift for the pyridyl ^1^H nuclei as:^43^
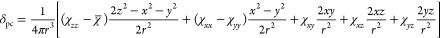
2where ). We note that the magnetic susceptibility tensor that we calculate here with CASSCF-SO assumes a thermodynamic equilibrium (i.e., Boltzmann) population of electronic states, but that the timescale for equilibration of the electron spins (*T*_1e_) is possibly longer than the 30 fs gap between points in our calculation. Although there are no direct measurements of *T*_1e_, estimates of Finney et al. for a series of Ln(III) complexes of DOTA-derivatives gave *T*_1e_ on the order of 300–800 fs.^44^ In fact, the question of the *T*_1e_ timescale is incredibly important for contrast agents, and is one that we will address in a follow-up to the present paper.

Herein we also determine the average molecular structure of the d-[GdL^1^] complex over the whole trajectory by rotating and aligning the molecular structure in each frame to the structure in the first frame,^45^ and then averaging the coordinates (Tables S2–S4). We have also performed a single-point CASSCF-SO calculation on this average structure with Dy(III) to determine the magnetic anisotropy and δ_pc_. These calculations do not include solvent, as it is non-sensical to average the solvent positions over the MD trajectory.

## Results and Discussion

**Figure 2:**
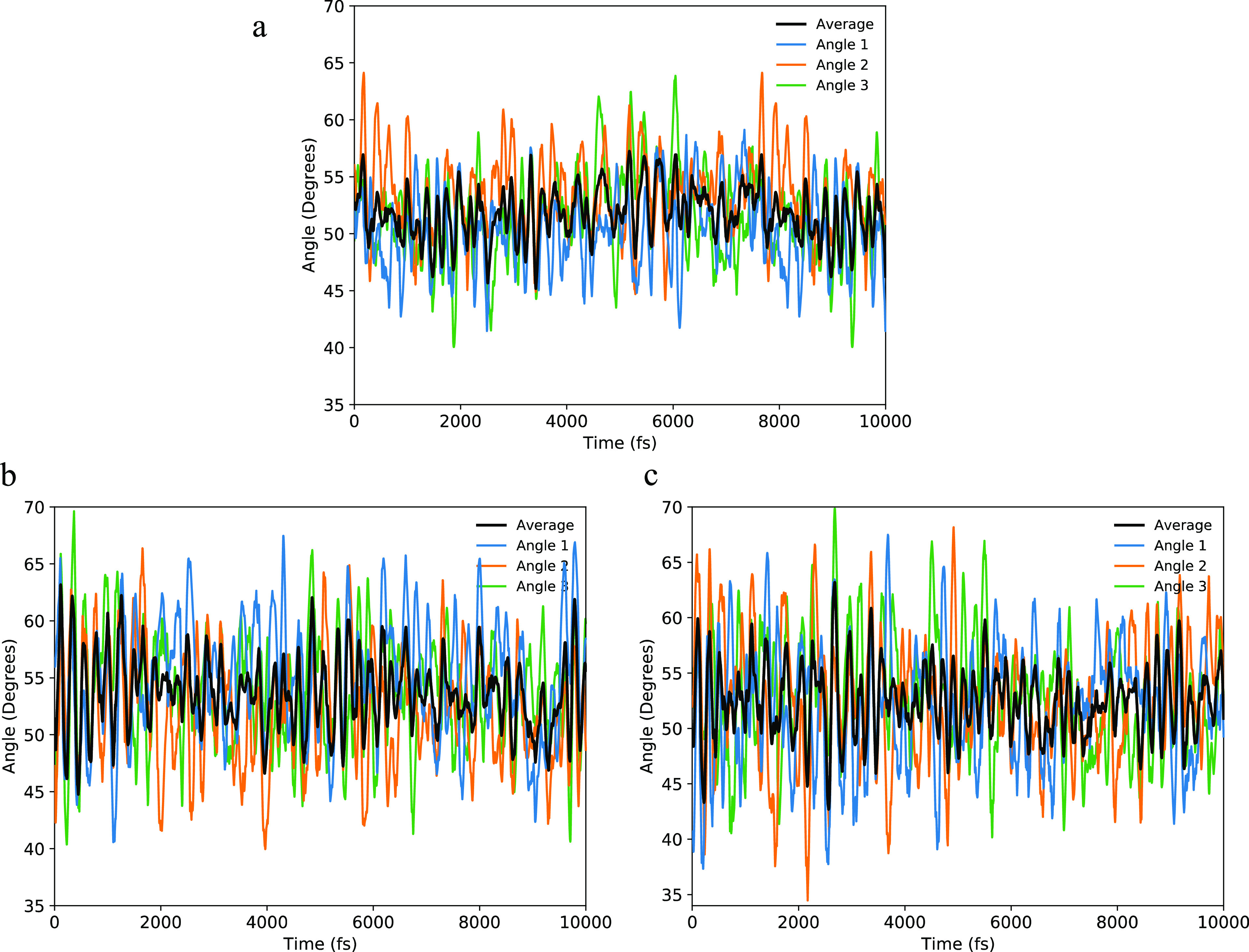
Time evolution of the O–Gd–C̃_3_ angles from AIMD simulations of d-[GdL^1^] in (a) D_2_O, (b) MeOD, and (c) d_6_-DMSO. Angles 1–3 are formed by the three “arms” of the ligand with the Gd ion and the C̃_3_ axis, i.e., O1–Gd–C̃_3_, O2–Gd–C̃_3_, and O3–Gd–C̃_3_ (cf. Figure 1).

AIMD simulations were performed on d-[GdL^1^] in D_2_O, MeOD, and d_6_-DMSO (“Methods,” Figures S1–S3). To obtain information on the molecular coordination geometry, we calculated the O–Gd–C̃_3_, N_eq_–Gd–C̃_3_, and N_ax_–Gd–C̃_3_ angles and examined the fluctuations over time. The C̃_3_ is defined here as the average of the vectors normal to the planes formed by (i) the three Gd-bonded oxygen atoms; (ii) the three triazacyclononane nitrogen atoms (N_ax_), and (iii) the three pyridine nitrogen atoms (N_eq_). The simulations show similar behavior in all three solvents, with average O–Gd–C̃_3_ angles of *ca.* 50°–55° and large oscillations of ±10°–20° (Table 1 and Figure 2). The average N_ax_–Gd–C̃_3_ angles are *ca.* 38–40 ± 7–14°, and the average N_eq_–Gd–C̃_3_ angles are *ca.* 90–91 ± 7–12° (Table S1, Figures S4–S6). Interestingly, the average O–Gd–C̃_3_ angle in MeOD is larger than in D_2_O, in agreement with our original average structure model used to interpret the experimental data, but the difference in the average angles in the MD simulations is *ca.* 3° compared to *ca.* 1° (Table 1).^6^ Furthermore, the average O–Gd–C̃_3_ angle in d_6_-DMSO from the MD simulations is *ca.* 0.9° smaller than that in MeOD, whereas the average structure model predicted it to be *ca.* 0.5° larger.^6^ Clearly, however, the oscillations observed during the trajectories are far larger than the tiny differences in the average angles, both in the AIMD simulations and our previous average structure models, making this comparison somewhat redundant.

**Table 1:** Minimum, Maximum, and Average O–Gd–C̃_3_ Angles Obtained from the AIMD Trajectories on d-[GdL^1^] in the Three Solvents^a^

solvent	minimum angle (°)	maximum angle (°)	average angle (°)	average angle (previous work) (°)^6^
D_2_O	40.1	64.1	51.8	52.0
MeOD	40.0	69.6	53.6	53.3
d_6_-DMSO	34.5	69.8	52.5	53.8

a Average angles from our previous work, using average structural models, are shown in for comparison.^6^

**Figure 3:**
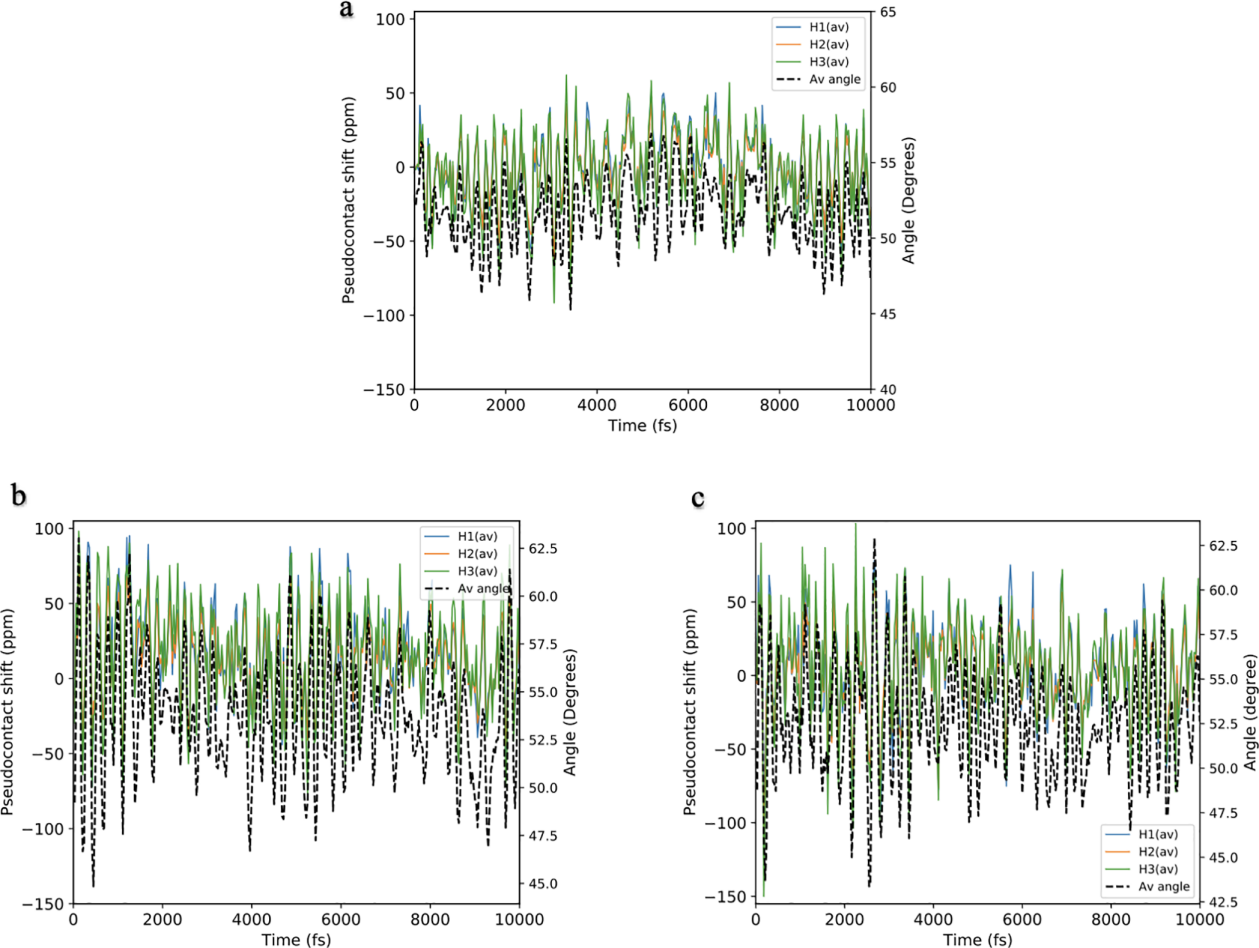
Average pseudocontact shift δ_pc_ (ppm) for each of the chemically-independent hydrogen atoms in the aromatic “arms” of the [DyL^1^] complex in (a) D_2_O, (b) MeOD and (c) d_6_-DMSO (blue, orange, and green respectively; c.f. Figure 1). The average O–Dy–C̃_3_ angles (in degrees) from the AIMD calculations are shown on the secondary axis and plotted as black dashed lines to show the strong correlation with the δ_pc_.

To obtain δ_pc_, we performed CASSCF-SO calculations on structural models extracted from every 30^th^ frame of the AIMD trajectories (i.e., every 30 fs), with this interval chosen to provide a good balance of computational cost and accuracy (Figure S7). The CASSCF-SO calculations directly yield the magnetic susceptibility tensor (Figures S8–S10), which we find has a consistent isotropic part in all three solvents of around 0.05 cm^3^ mol^–1^ at 298 K, but an anisotropy that fluctuates substantially. Notably, across all solvents, the tensor is most often closest to easy-axis anisotropy (one large and two smaller eigenvalues), but fluctuations can sometimes make the tensor closer to easy-plane anisotropy (two large and one small eigenvalue); however, the tensor is nearly always non-axial. These data, in conjunction with the atomic coordinates, then allow us to calculate δ_pc_ (eq 2; Figure 3). In the present case, we are interested in the δ_pc_ of the pyridyl H atoms (H_1_–H_3_ in Figure 1) because these are sufficiently distant from the Ln(III) ion that δ_c_ is negligible, and because these signals were the basis of the structural interpretation in our previous study.^6^ Due to the explicit solvent dynamics in the AIMD simulations, the complex has no symmetry, and thus all the H atoms are inequivalent (Figures S11–S13), but for simplicity, we also present the average δ_pc_ of each triplet of H atoms that are equivalent in the symmetric complex (Figure 3, c.f. Figure 1). We find that the oscillations in δ_pc_ are very large, on the order of ±80, ±90, and ±130 ppm in D_2_O, MeOD, and d_6_-DMSO, respectively (Table 2). We also observe that the shifts for each H_1_, H_2_, and H_3_ are correlated with one another, suggesting that the changes are driven by the magnetic anisotropy (although this is not separable from the structural part in eq 2). Interestingly, the calculated δ_pc_ is also correlated with the average O–Dy–C̃_3_ angle in the D_2_O and d_6_-DMSO simulations, but seemingly less so in the MeOD simulations.

**Table 2:** Comparison of the Experimental^6^ Pseudocontact Shifts δ_pc_ of the Pyridyl Protons in [DyL^1^] to Time-Averaged Values Calculated Using AIMD and CASSCF-SO Calculations, Values Calculated Using the Averaged Structure from AIMD Trajectories, and Values Obtained Based on DFT-Optimized Structures^6^

	D_2_O				MeOD			d_6_-DMSO		
	exp. (ppm)	calculated			exp. (ppm)	calculated		exp. (ppm)	calculated	
		time-avg. (ppm)	avg. struct. (ppm)	DFT opt.^a^ (ppm)^6^		time-avg. (ppm)	avg. struct. (ppm)		time-avg. (ppm)	avg. struct. (ppm)
H_1_	2.9	–1.4	44.1	–19.3	16.2	16.0	37.1	21.6	4.1	59.6
				7.8						
				–33.3						
H_2_	2.4	–1.9	30.4	–15.3	13.6	13.5	19.4	18.0	3.6	–25.3
				6.0						
				–24.8						
H_3_	1.7	–3.2	119.5	–17.7	16.6	17.5	26.9	22.3	4.9	–94.5
				6.6						
				–25.4						
range	1.2	1.8			3	4		4.3	1.3	
max.		62.1				98.2			103.2	
min.		–91.7				–79.2			–150.0	

a DFT optimized data obtained using M06/SMD, M06/PCM, and BP86/SMD methods, respectively, taken from ref (6).

The same raw data for the individual H atoms (Figures S11–S13) can also be used to construct histogram spectra for the protons of interest (Figures S14 and S15), which show that H_2_ is the least affected by the structural dynamics (as it has a comparatively narrow distribution), while both H_1_ and H_3_ have far broader and less symmetric distributions; this is true for all three solvents. However, given that the collection time of the free-induction decay for ^1^H NMR spectra is usually on the timescale of 0.1–10 s, to compare with the experiment, we should average over the whole of our trajectory (10 ps). These time-averaged δ_pc_ values (Table 2), show spectacularly good agreement with experiment for the MeOD simulation, with a root-mean-square deviation (RMSD) between the experimental and time-averaged calculated values of only 0.5 ppm: this is remarkable given the ±90 ppm fluctuations in the calculations. While the agreement between the experiment and the D_2_O and d_6_-DMSO simulations is less good, especially in the case of d_6_-DMSO, we note that the ordering of the calculated time-average δ_pc_ values match the experimental ordering of H_1_ > H_2_ > H_3_ in D_2_O and H_3_ > H_1_ > H_2_ in MeOD and d_6_-DMSO, and that the ranges of δ_pc_ spanned by the three protons are in good agreement in all cases (Table 2). We posit that the less good agreement between theory and experiment in d_6_-DMSO could occur because it is the most complex of the solvents employed here, and thus its dynamics are more nuanced than those of D_2_O and MeOD. We note that our AIMD trajectories are far shorter than the timescale of the experiment, and so the obtained agreement is excellent given this limitation.

Another possible method to distill the AIMD data into δ_pc_ could be by calculating the time-average molecular structure (see “Methods”) and using this to calculate δ_pc_. However, this approach gives very poor results compared to the experiment, which mimics our previous findings with DFT optimizations (Table 2);^6^ this is a consequence of the hypersensitivity of the magnetic anisotropy to the molecular structure and underscores the need for molecule-level structural information in this case. Hence, despite the fact that our ab initio AIMD + CASSCF-SO method for calculating δ_pc_ entails several notable approximations [in particular: (i) AIMD trajectories examine one molecule over picoseconds, compared to *ca.* 10^19^ molecules over seconds in the experiment; (ii) we use dispersion-corrected DFT to describe the intra- and intermolecular interactions and hence the structural dynamics; (iii) we use minimal CASSCF-SO calculations to obtain the magnetic anisotropy and assume fast electron spin relaxation times (*T*_1e_); (iv) we neglect all contact (spin delocalization) contributions to the paramagnetic shift], we find good agreement with experiment, and we are therefore confident in our qualitative conclusion that the structure and pseudocontact shifts show large fluctuations in time and are strongly correlated with one another. The molecule-level detail obtained herein thus highlights large conceptual differences from the average structure model employed in our previous study, and indeed commonly in the literature.

## Conclusions

We have used DFT-based AIMD calculations to study the structure of a prototypical Gd(III) PARASHIFT agent in D_2_O, MeOD, and d_6_-DMSO. The simulations show drastic oscillations in the O–Gd–C̃_3_ angles, and indicate that previous results based on an average structural model do not capture a significant feature of the molecule–solvent interactions. In these compounds, where the magnetic anisotropy is exquisitely sensitive to the molecular structure, the large structural variations as a function of time result in large fluctuations in the pseudocontact shift for [DyL^1^], as an exemplar member of the family, of around ±80, ±90, and ±130 ppm in D_2_O, MeOD, and d_6_-DMSO, respectively, which is quite astounding given that the average experimental shifts are only 2, 15, and 21 ppm, respectively. Thus, the AIMD + CASSCF-SO approach developed and applied here provides an important conceptual advance in our understanding of the behavior of [LnL^1^] in solution. We expect that these drastic local fluctuations in structure and magnetic anisotropy will have profound consequences for the electron spin dynamics, and hence the nuclear spin dynamics, and work is currently underway to investigate this further.

## Supporting Information Available

Energy and pressure during AIMD trajectories; minimum, maximum and average N_ax_–Gd–C̃_3_ and N_eq_–Gd–C̃_3_ angles; time-dependence of N_ax_–Gd–C̃_3_ and N_eq_–Gd–C̃_3_ angles; time-dependence of magnetic susceptibility tensor principal values; time-dependence of all δ_pc_ for each proton of interest; histogram spectral representations of δ_pc_; atomic coordinates of time-averaged structures (PDF)
