# Supplementary material for: Structural Evolution of Paramagnetic Lanthanide Compounds in Solution Compared to Time- and Ensemble-Average Structures
Source: J Am Chem Soc. 2023 Jun 16;145(25):13632–9. doi: 10.1021/jacs.3c01342 (PMC10311533; doi:10.1021/jacs.3c01342)
Supplement: Supplementary file 1 — ja3c01342_si_001.pdf [file ja3c01342_si_001.pdf]

Supporting Information

Structural evolution of paramagnetic lanthanide compounds in solution compared to time- and ensemble-average structures

Barak Alnami, Jon G. C. Kragskow, Jakob K. Staab, Jonathan M. Skelton* and Nicholas F. Chilton*

Department of Chemistry, The University of Manchester, Manchester, M13 9PL, UK

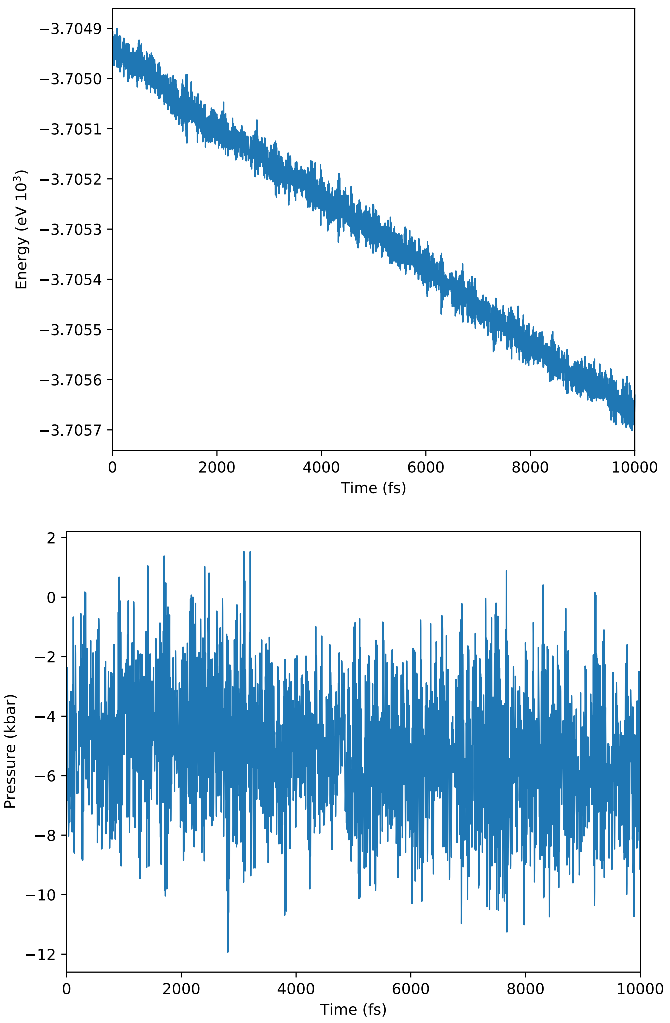

Figure S1. Energy (top) and pressure (bottom) as a function of time during the production AIMD simulation on d-[GdL^1] in D_2O.

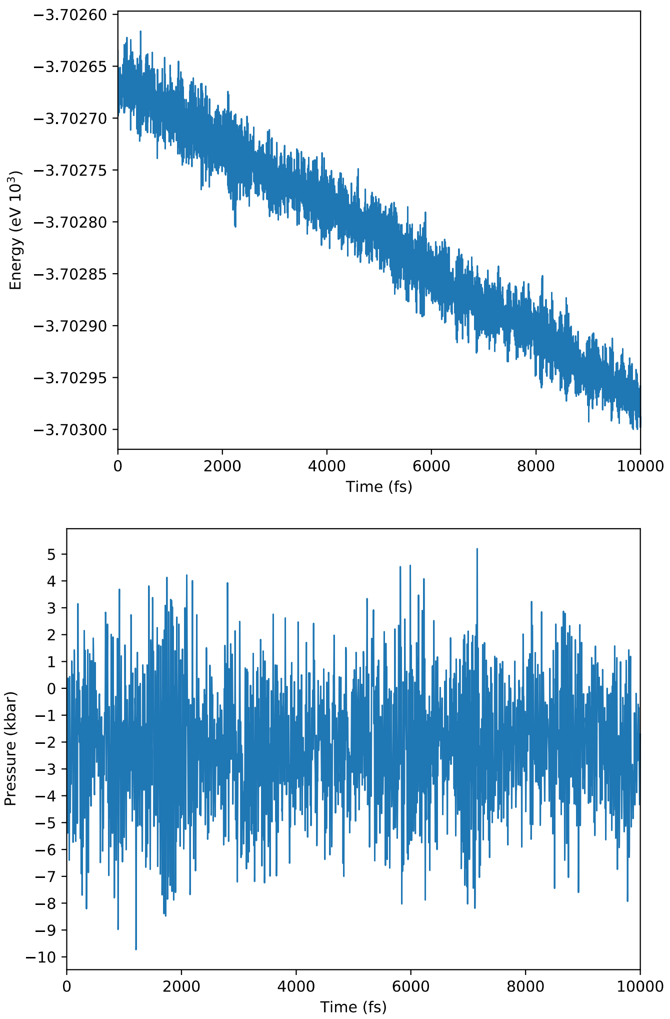

Figure S2. Energy (top) and pressure (bottom) as a function of time during the production AIMD simulation on d-[GdL[1]] in MeOD.

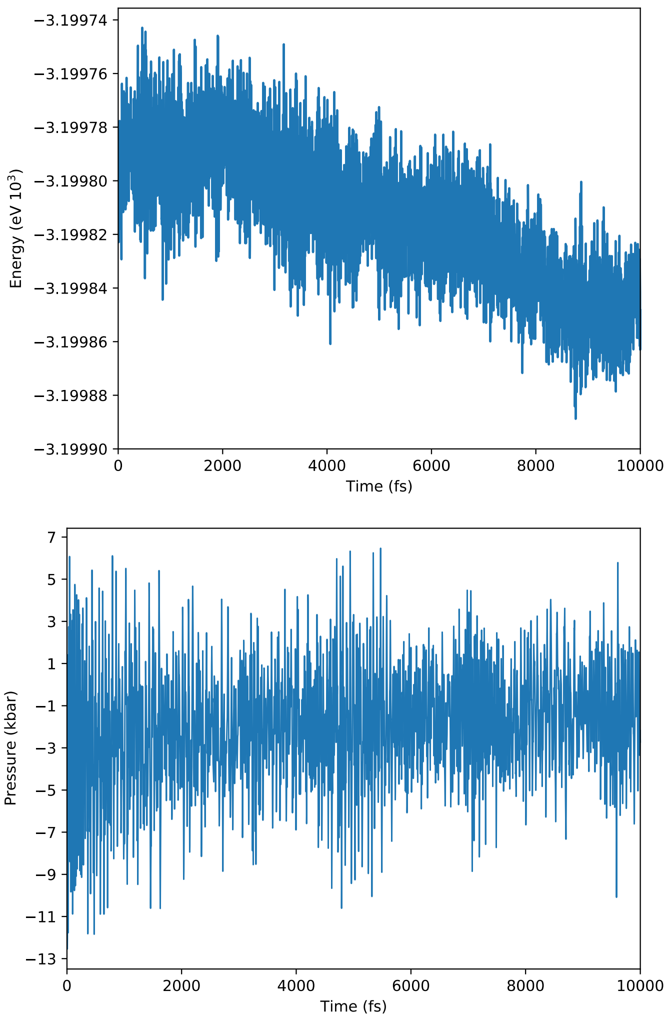

Figure S3. Energy (top) and pressure (bottom) as a function of time during the production AIMD simulation on d-[GdL[1]] in d_6-DMSO.

Table S1. Minimum, maximum and average N_{ax}-Gd-$\tilde{C}_3$ and N_{eq}-Gd-$\tilde{C}_3$ angles obtained from the AIMD trajectories on d-[GdL[1]] in the three solvents.

	**D_2O**		**MeOD**		**d_6-DMSO**	
	**N_{ax} (°)**	**N_{eq} (°)**	**N_{ax} (°)**	**N_{eq} (°)**	**N_{ax} (°)**	**N_{eq} (°)**
**Minimum**	33.7	84.6	29.7	83.3	23.6	79.0
**Maximum**	47.0	97.8	46.7	96.8	50.4	101.9
**Average**	39.6	91.1	38.7	90.0	38.2	89.9

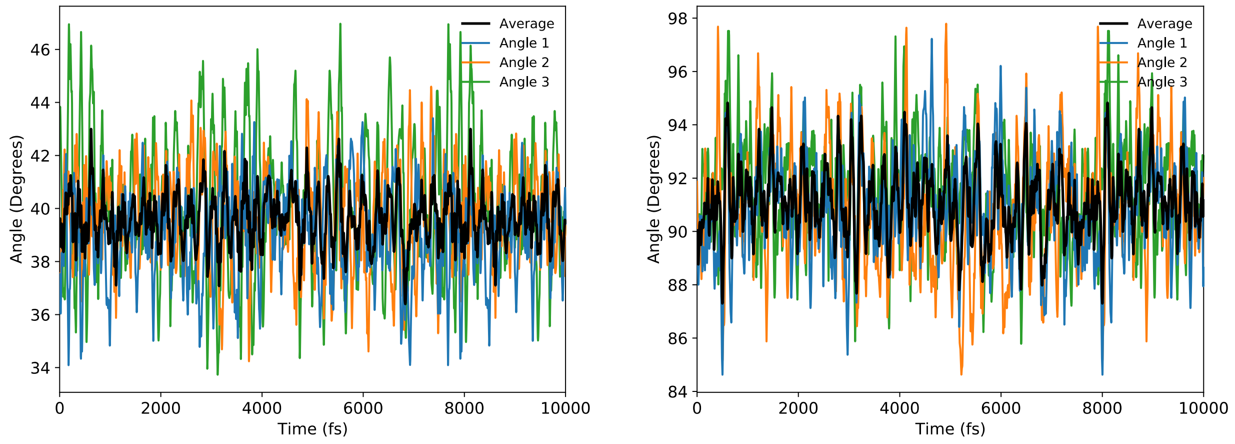

Figure S4: Time evolution of the N_{ax}-Gd-$\tilde{C}_3$ angle (left) and the N_{eq}-Gd-$\tilde{C}_3$ angle (right) from AIMD simulations of d-[GdL[1]] in D_2O.

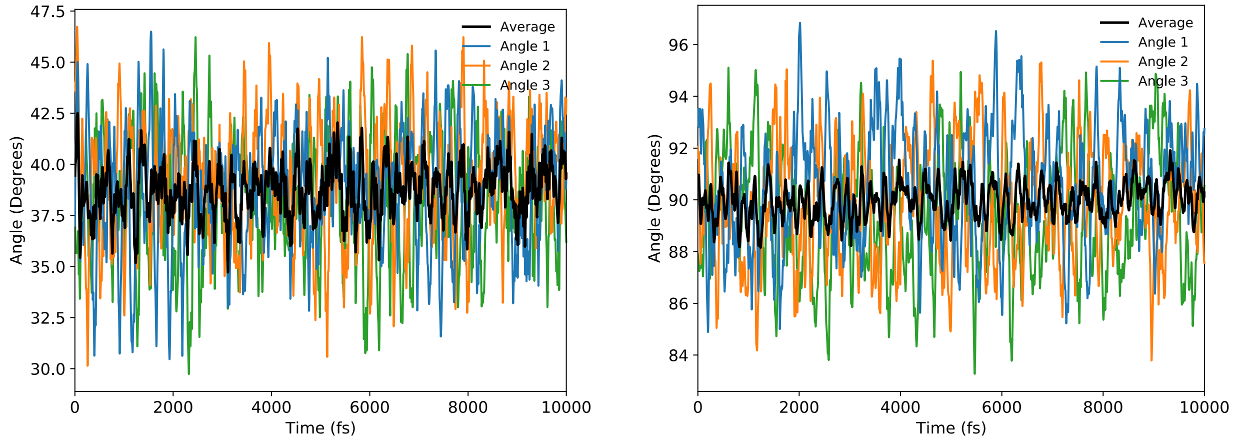

Figure S5: Time evolution of the N_{ax}-Gd-$\tilde{C}_3$ angle (left) and the N_{eq}-Gd-$\tilde{C}_3$ angle (right) from AIMD simulations of d-[GdL[1]] in MeOD.

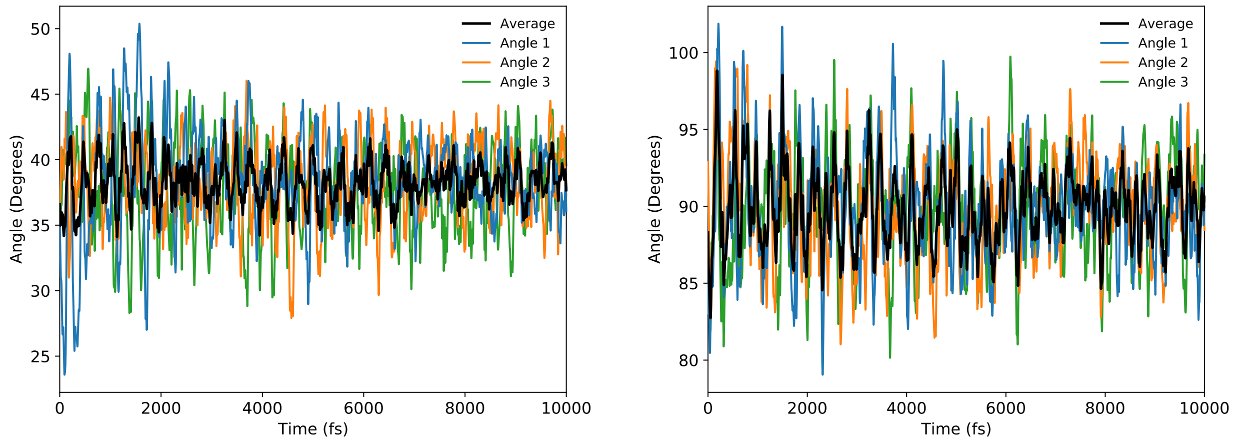

Figure S6: Time evolution of the N_{ax}-Gd-$\tilde{C}_3$ angle (left) and the N_{eq}- Gd-$\tilde{C}_3$ angle (right) from AIMD simulations of d-[GdL^1] in d_6-DMSO.

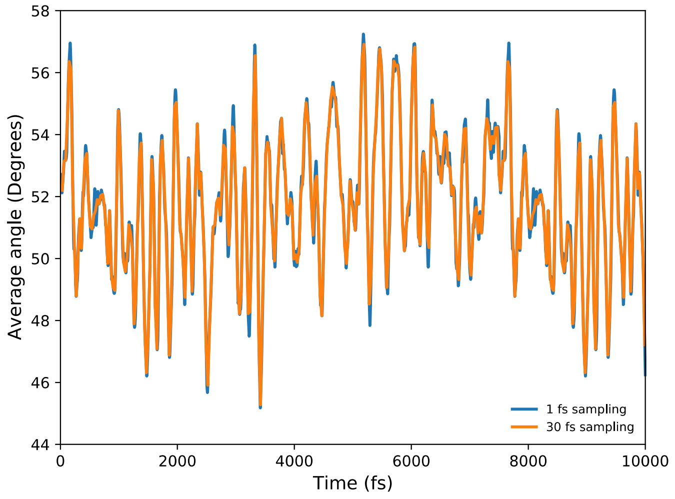

Figure S7: Time evolution of the average O-Gd-$\tilde{C}_3$ angle from AIMD simulations of d-[GdL^1] in D_2O taken every 1 fs (blue) and every 30 fs (orange).

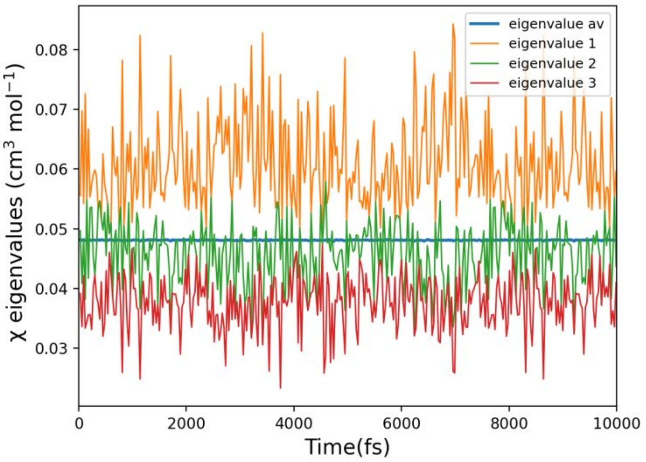

Figure S8: Time evolution of the eigenvalues of the magnetic susceptibility tensor at 293 K for [DyL[1]] in D_2O. Eigenvalues are ordered such that $\chi_1 > \chi_2 > \chi_3$.

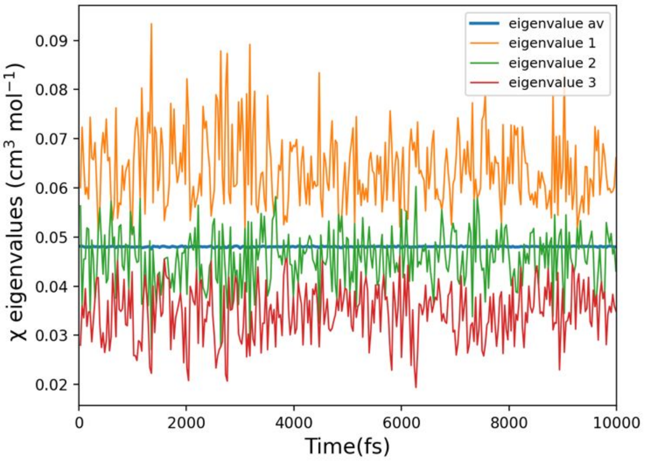

Figure S9: Time evolution of the eigenvalues of the magnetic susceptibility tensor at 293 K for [DyL[1]] in MeOD. Eigenvalues are ordered such that $\chi_1 > \chi_2 > \chi_3$.

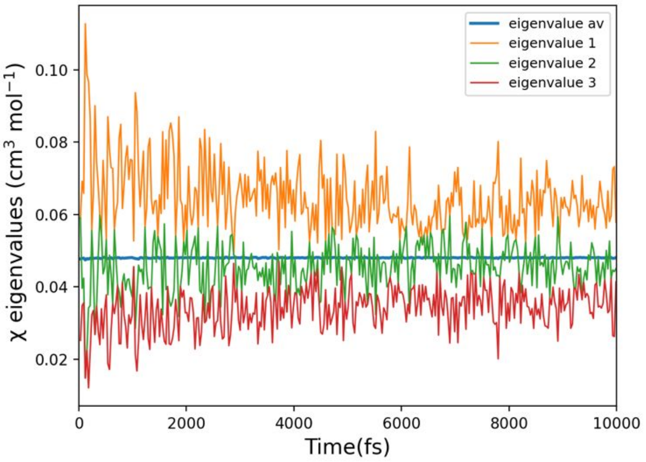

Figure S10: Time evolution of the eigenvalues of the magnetic susceptibility tensor at 293 K for [DyL[1]] in d_6-DMSO. Eigenvalues are ordered such that $\chi_1 > \chi_2 > \chi_3$.

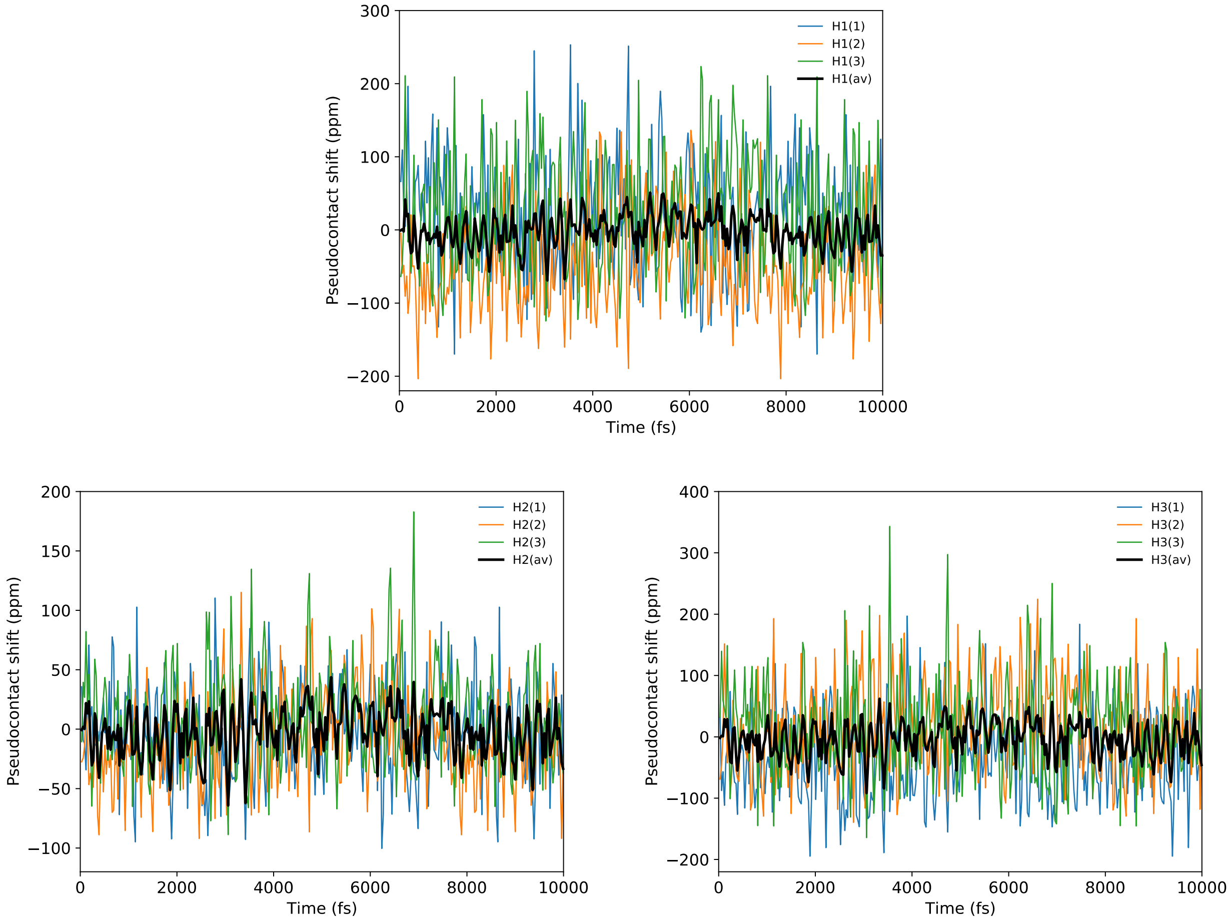

Figure S11. Pseudocontact shift δ_{pc} (ppm) as a function of time for the three triplets of nominally equivalent H_1, H_2 and H_3 protons in each "arm" of the ligand in [DyL[1]] in D_2O.

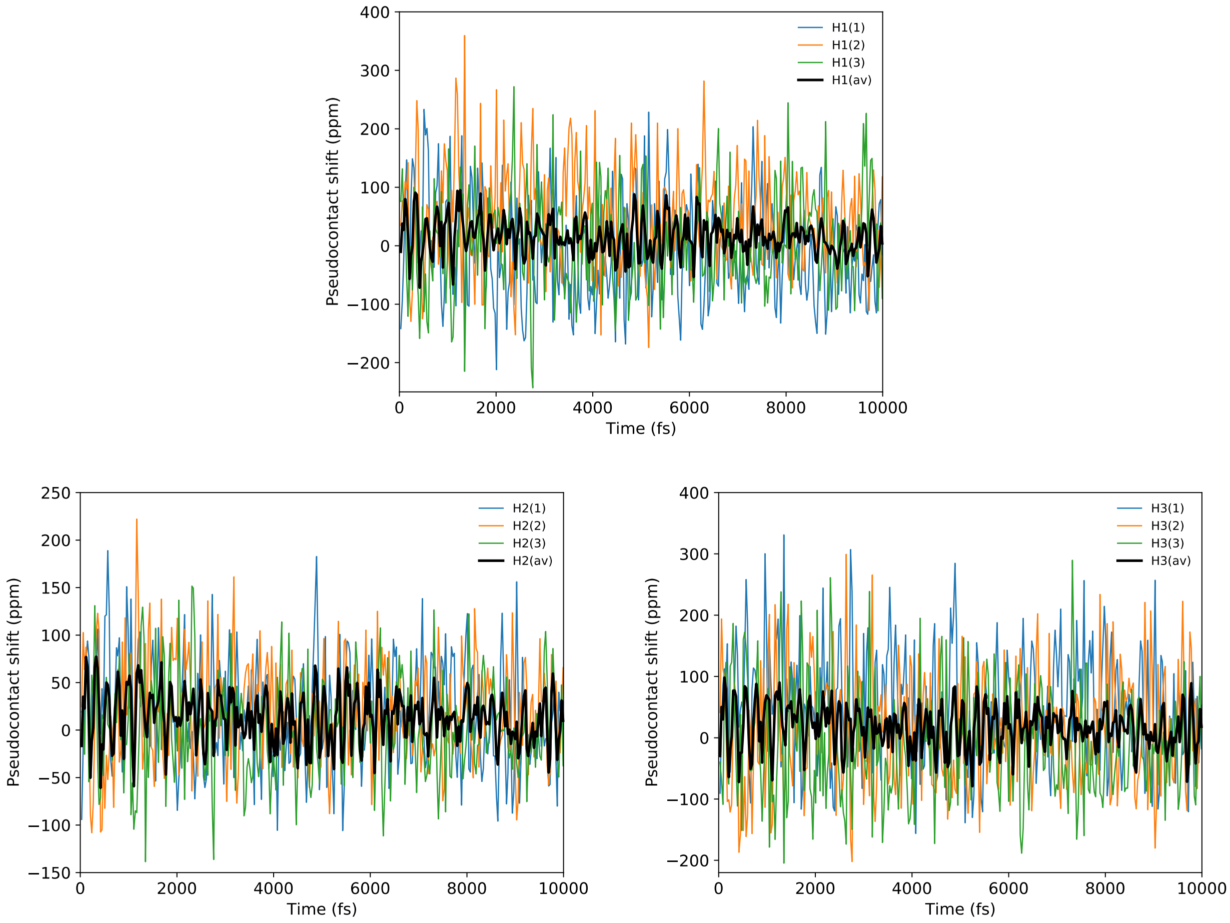

Figure S12. Pseudocontact shift δ_{pc} (ppm) as a function of time for the three triplets of nominally equivalent H_1, H_2 and H_3 protons in each "arm" of the ligand in [DyL^1] in MeOD.

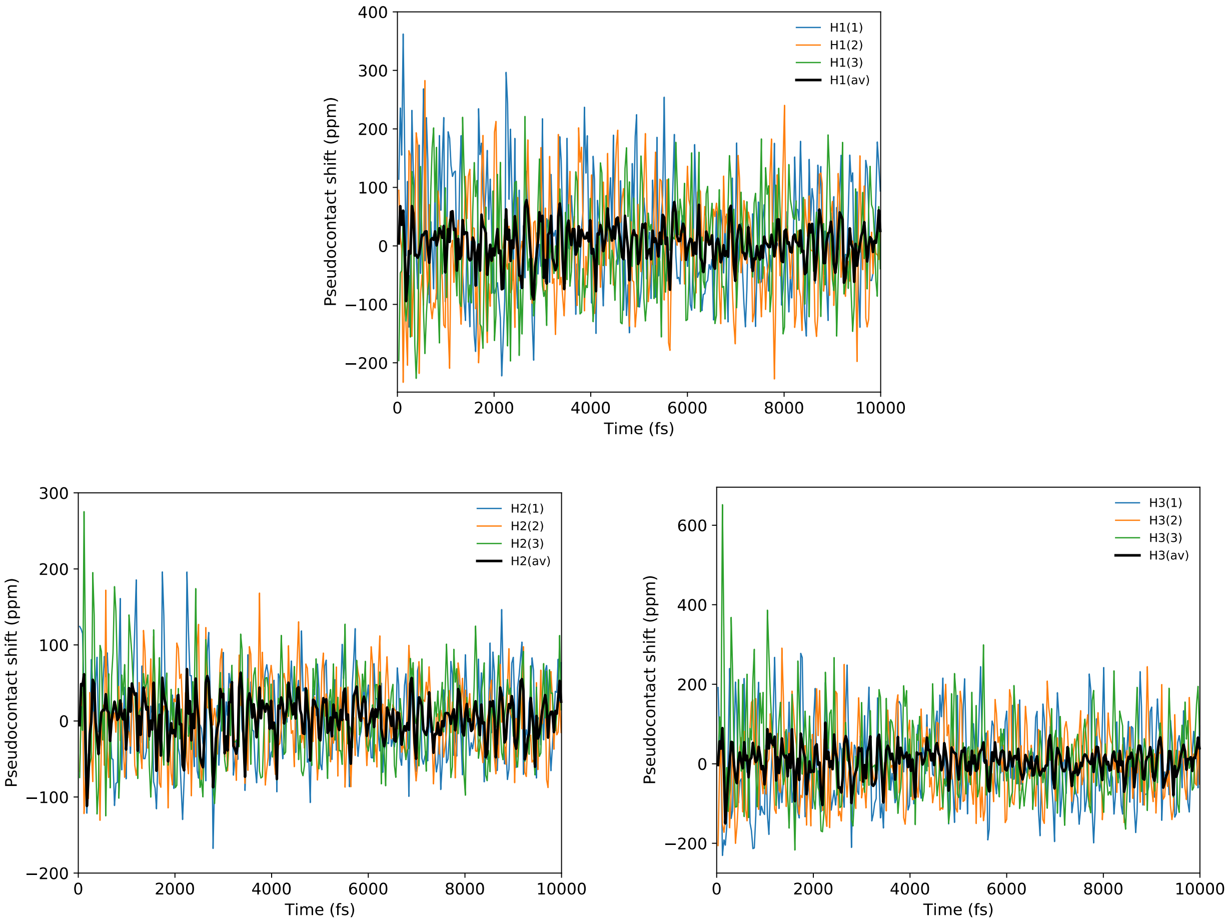

Figure S13. Pseudocontact shift δ_{pc} (ppm) as a function of time for the three triplets of nominally equivalent H_1, H_2 and H_3 protons in each "arm" of the ligand in [DyL^1] in d_6-DMSO.

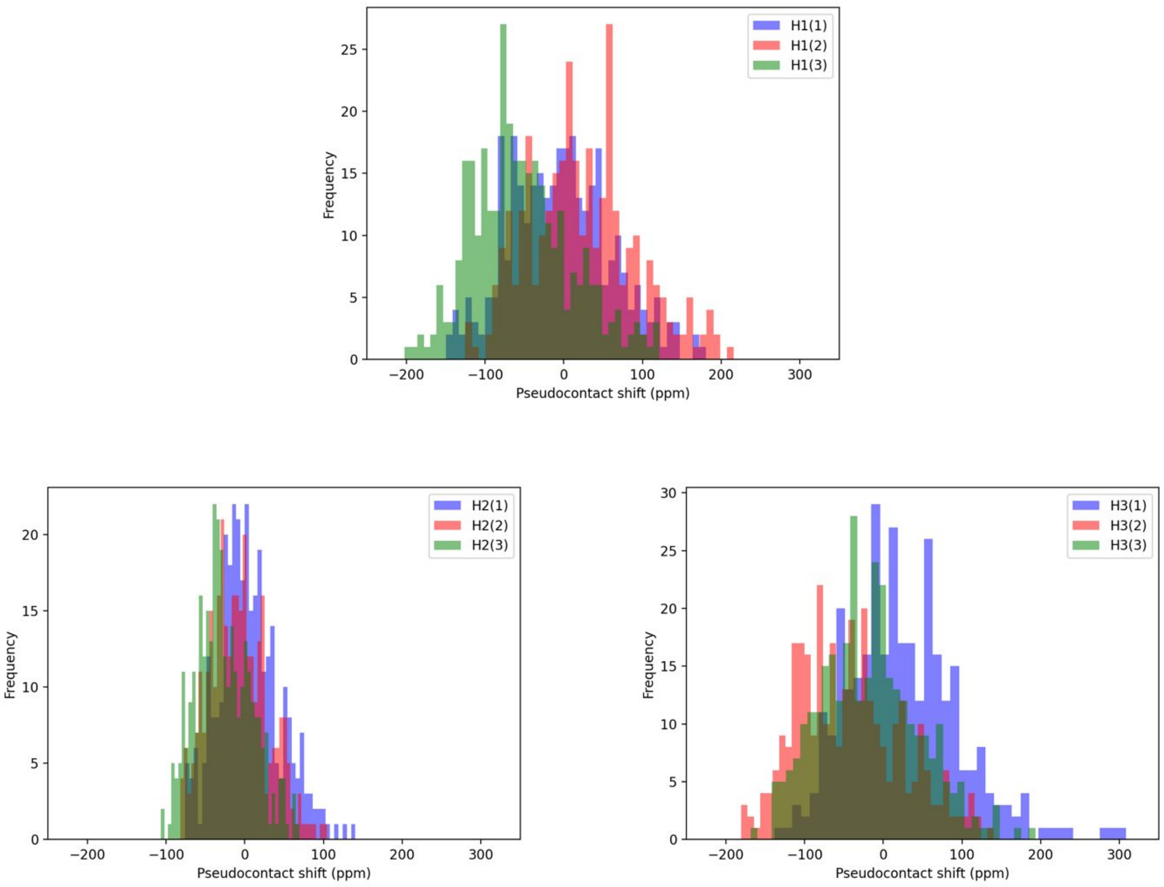

Figure S14. Spectral representation of the pseudocontact shifts δ_{pc} (ppm) for the three triplets of nominally equivalent H_1, H_2 and H_3 protons in each "arm" of the ligand (given in parentheses) in [DyL^1] in D_2O.

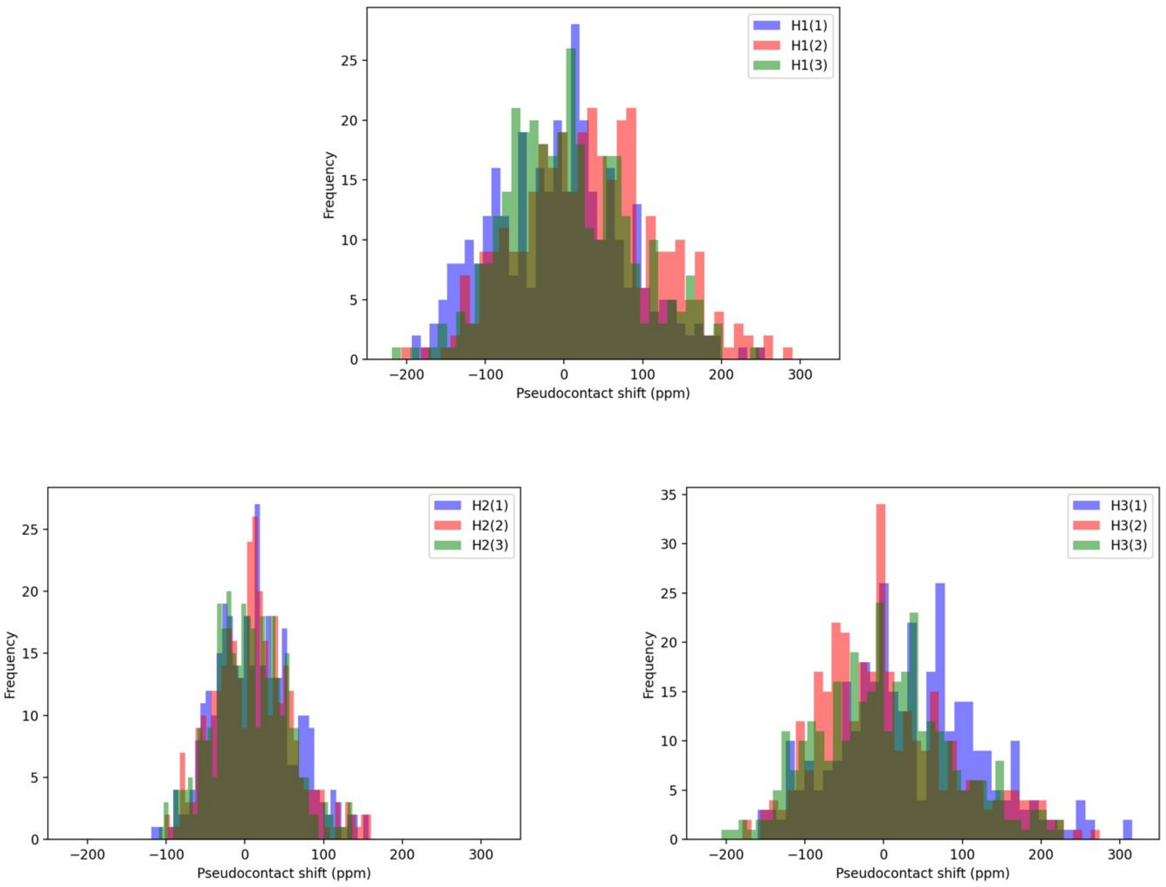

Figure S15. Spectral representation of the pseudocontact shifts δ_{pc} (ppm) for the three triplets of nominally equivalent H_1, H_2 and H_3 protons in each "arm" of the ligand (given in parentheses) in [DyL^1] in MeOD.

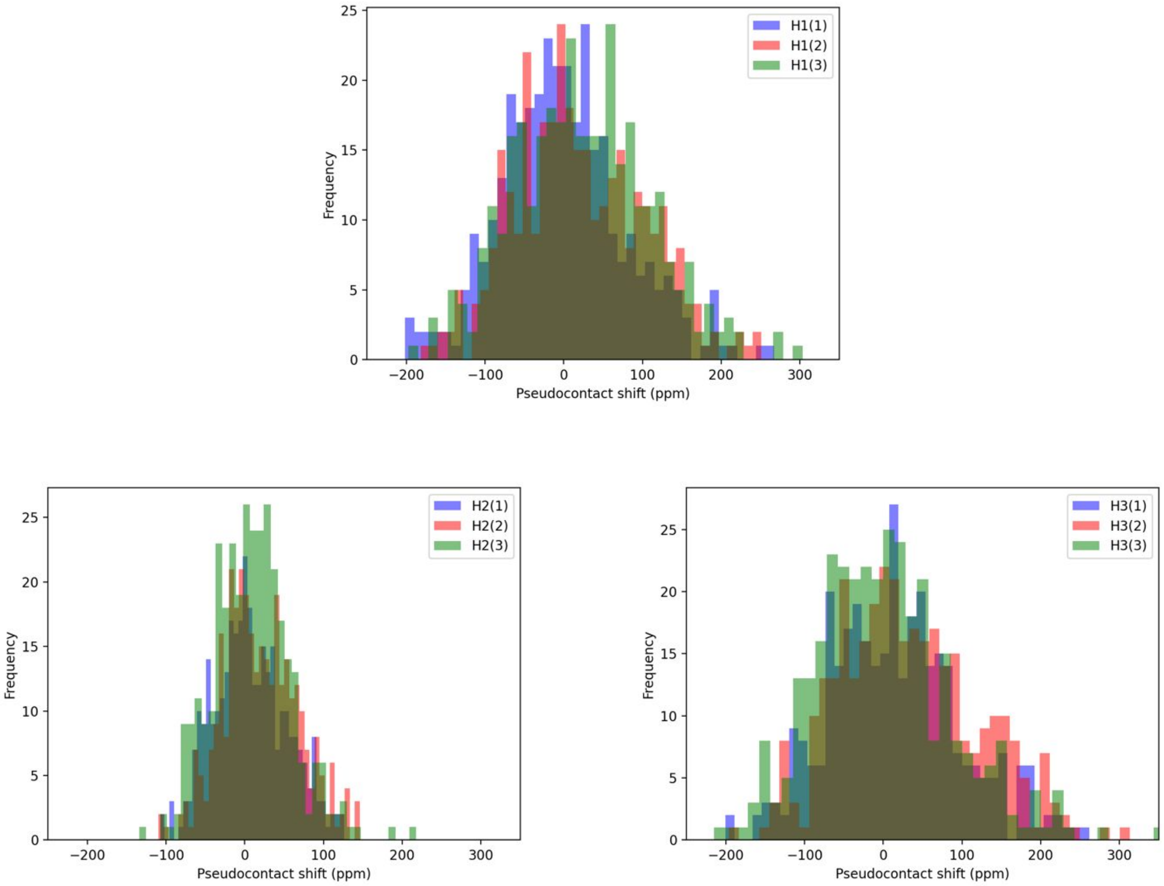

Figure S16. Spectral representation of the pseudocontact shifts δ_{pc} (ppm) for the three triplets of nominally equivalent H_1, H_2 and H_3 protons in each "arm" of the ligand (given in parentheses) in [DyL^1] in d_6-DMSO.

Table S2. Atomic coordinates of the average structure of d-[GdL^1] from AIMD simulations in D_2O.

Atom	x (Å)	y (Å)	z (Å)
O	1.3852577	0.9359823	1.6241697
O	2.6774568	0.7018642	3.6780985
O	1.2714843	0.9425552	-1.7011071
O	1.8990557	2.5501705	-2.9861994
O	1.3651685	-1.9395863	-0.2637818
O	1.8746473	-4.135428	-0.964502
C	-2.8073236	-0.7232149	1.6015242
C	-2.4053595	0.6436967	2.2730893
C	-2.7008203	2.053015	0.1650629
C	-2.2534916	1.8463821	-1.441591

C	-2.8673134	-0.5508869	-1.5770826
C	-2.4531732	-1.7540372	-0.8065031
C	-1.3467705	-2.649732	1.2461045
C	-0.6708688	-2.1044621	2.4225139
C	-0.874967	-2.6380978	3.7205299
C	-0.1239524	-2.1442149	4.703811
C	0.764108	-1.1763514	4.4624117
C	0.873708	-0.674698	3.1727883
C	1.7738668	0.3632322	2.8047553
C	-1.3925038	2.7811628	2.0429821
C	-0.420398	3.4015015	1.1205917
C	-0.13847	4.7432518	1.4169419
C	0.7733339	5.2668011	0.5211048
C	1.3395319	4.3199356	-0.5184731
C	0.9729752	3.0076679	-0.7033669
C	1.4753295	2.0677782	-1.8941723
C	-1.3428452	0.3137213	-3.0855791
C	-0.6006458	-1.0012073	-3.3488531
C	-0.4163768	-1.855671	-4.5706819
C	0.2868447	-3.0745139	-4.6866858
C	0.860361	-3.5009562	-3.5568199
C	0.6745585	-2.6607213	-2.3179488
C	1.3985533	-2.9886388	-1.1082659
N	-1.8518471	-1.421175	0.5649503
N	-1.8278966	1.5072851	1.2700267
N	-1.8382743	0.4339957	-1.7075995
N	0.1130339	-1.0862946	2.189054
N	0.119068	2.5265081	0.1486438
N	-0.0571047	-1.4808062	-2.1742246
Gd	0	0	0
H	-3.003546	-1.459806	2.427488
H	-3.6629188	-0.5896752	1.1084287
H	-3.2042684	1.2480816	2.7620498
H	-1.7241018	0.4271054	3.0665227
H	-2.888316	3.1138805	0.3826835
H	-3.5647776	1.6318923	0.2725316
H	-3.0225986	2.0767463	-2.0835658
H	-1.4973077	2.5850193	-1.781768
H	-3.1278046	-1.0050907	-2.5391568
H	-3.7132495	0.0025289	-1.1732212
H	-3.2423465	-2.2608667	-0.7714294

H	-1.7857109	-2.4860834	-1.322422
H	-0.8110451	-3.2443657	0.4588308
H	-2.2061469	-3.1559722	1.6167143
H	-1.4871225	-3.5340708	3.9368246
H	-0.2665031	-2.508805	5.6884157
H	1.4093578	-0.8927844	5.2741371
H	-0.8940505	2.4225656	2.8913004
H	-2.0554977	3.6192314	2.3731233
H	-0.6183145	5.446468	2.2442897
H	1.0079425	6.3372594	0.7202663
H	2.0769705	4.6284635	-1.2254649
H	-2.1123697	0.4922961	-3.8933837
H	-0.644299	1.1911766	-3.3215264
H	-0.9111448	-1.4171256	-5.4794148
H	0.4129518	-3.7321592	-5.6387443
H	1.4484038	-4.3823898	-3.680807

Table S3. Atomic coordinates of the average structure of d-[GdL^1] from AIMD simulations in MeOD.

Atom	x (Å)	y (Å)	z (Å)
O	0.8381054	1.5411295	1.5096238
H	-0.6006871	3.8208405	0.0184041
O	2.1143755	-0.3197898	-0.6653138
O	4.2339292	0.0990072	-1.2545027
O	0.5597913	-2.0716874	1.2408152
O	0.6575819	-4.2715183	1.9541666
H	-0.6553963	-1.9483063	-3.2502606
H	0.8274124	-1.063673	-2.6745059
H	0.5187418	-4.3820827	-3.4753899
H	1.128285	-6.3795734	-2.2521786
H	-1.2368762	2.0710741	4.5315111
H	-0.8147206	4.3242154	-1.3211456
H	0.9652035	-5.9908667	0.1931418
H	1.4000564	5.5944915	-1.9574393
H	3.858363	5.2791852	-2.5511152
H	4.6403283	2.8273771	-2.062966
O	1.1999717	2.5211668	3.0513884
H	-2.7455869	1.4561445	-2.0148532
C	-3.7613211	0.5823357	0.0554578
C	-2.7285296	2.0143148	-0.1435713

C	-1.7038494	1.7834468	-2.0039037
C	-0.9269231	0.703085	-2.6787553
C	-2.778148	-1.1995596	-1.8143856
C	-3.284222	-1.7576115	-0.5889054
N	-1.3944088	2.0373095	-0.8927077
C	-2.6435136	-0.6971283	2.437718
N	-1.4002657	0.0371015	2.1609425
N	1.3905311	1.9777633	-0.9979217
N	0.1013034	-2.6097031	-0.7641848
C	-0.9088812	0.9902583	2.912809
C	0.4637962	1.7723438	2.4977038
C	-0.5574536	3.4266491	-0.7588277
C	0.88671	3.3539948	-1.208247
C	1.7527777	4.5566912	-1.7580655
C	3.0927302	4.3628534	-2.0344987
C	3.6396464	3.0168354	-1.8915581
C	2.7019463	1.8463113	-1.318328
C	2.9927762	0.4697402	-1.0254347
C	-0.3312357	-1.522798	-2.4852771
C	0.0817365	-2.8329855	-1.9082522
C	0.3984197	-4.1471417	-2.4688724
C	0.7877555	-5.2962467	-1.7934404
C	0.7649984	-5.1025665	-0.4954309
C	0.4924191	-3.717561	-0.1062461
C	0.5762718	-3.3135762	1.1161275
N	-2.9773859	-0.777674	0.3348839
C	-3.281673	-1.5355749	1.517583
N	-1.1512456	-0.6203849	-1.9865096
C	-3.4805103	-0.5363272	3.5657497
C	-2.8773391	0.3675889	4.3307129
C	-1.6029099	1.2266633	3.9562622
H	-4.5804599	0.6760824	0.644254
H	-4.2892641	0.464859	-0.721672
H	-3.4939692	2.8610521	-0.3432366
H	-2.2494444	2.131185	0.6614818
H	-1.4129069	2.8142992	-2.5150614
Gd	0	0	0
H	-1.0976397	0.6530802	-3.8180384
H	0.2511687	0.9033388	-2.7369252
H	-3.1953712	-1.9282437	-2.2205961
H	-3.3683363	-0.3719308	-2.3104352

H	-4.5370731	-1.9973214	-0.5563892
H	-2.7266244	-2.6226379	-0.4546714
H	-2.6168646	-2.4574621	1.2911029
H	-4.2257612	-2.1134161	1.8263645
H	-4.575646	-0.9944114	3.7218601
H	-3.4428745	0.4730285	5.1690097

Table S4. Atomic coordinates of the average structure of d-[GdL[1]] from AIMD simulations in d_6-DMSO.

Atom	x (Å)	y (Å)	z (Å)
H	0.0256334	-3.1611113	-6.1693383
H	0.9968286	-3.9734681	-4.2409166
H	-1.3306572	-0.944422	-5.3877452
H	-2.0729981	1.2338302	-3.974525
H	-0.4808855	1.6856551	-3.3756304
H	-2.9608084	-0.7606664	-2.6235777
H	-3.6830288	1.0714977	-1.9946886
H	-3.1047561	2.8563254	-1.8726836
H	-1.2818669	3.249632	-1.9141104
H	-5.0016964	-0.7245351	-0.760413
H	-3.7337715	-1.4411647	-0.8179758
H	-2.4709505	3.8334754	-0.1387292
H	-3.8998191	2.5567492	0.0876905
H	-4.0715291	-0.289607	1.9753184
H	-4.6993386	0.7516545	0.7754826
H	-2.4535314	-2.5238689	0.3924115
H	-3.7075506	-2.4902698	1.37965
H	-3.1764036	1.6742548	2.3113875
H	-1.6389731	0.729874	2.3312853
H	-1.5371286	3.6375742	2.1111695
H	-0.3859195	2.4105714	2.3422469
H	-2.8432918	-3.6697763	3.6186659
H	0.4726792	4.8736147	1.8999073
H	-1.5491894	-3.073267	5.3267218
H	2.3873173	5.8023106	0.3986402
H	0.7678695	-1.4923191	4.7474871
H	3.243854	4.6410351	-1.3560511
C	-0.1223965	-2.5761418	-5.0405182

C	0.4055613	-3.080703	-3.9326434
C	0.9631401	-2.8929333	-1.2858428
C	-0.8118602	-1.3295914	-4.6889815
C	0.2546371	-2.2988625	-2.5625221
C	-0.7584046	-0.5230315	-3.5355193
C	-1.3120742	0.9256942	-3.2671838
C	-3.0771917	0.1698553	-1.9086357
C	-2.2504744	2.5130502	-1.4905356
C	-3.6926235	-0.7219531	-0.8237767
C	-2.6621398	2.7564292	-0.1371728
C	-3.6396132	0.2978882	1.1722792
C	-2.6750367	-2.0790346	1.0780863
C	-2.5467305	1.2003725	1.7359109
C	-0.9133579	2.9695674	1.5680079
C	-1.5876291	-2.1570057	2.2222968
C	0.3021645	3.4491158	0.7441879
C	-1.8223585	-3.0064723	3.3995838
C	0.8456112	4.4208119	1.1257609
C	-1.1740667	-2.6264826	4.3891796
C	0.284101	-1.0443929	2.9772684
C	1.8858242	2.9451729	-0.7225538
C	1.8073675	4.9604261	0.3692427
C	-0.0096241	-1.6908924	4.1385504
C	1.5129704	-0.1967844	2.7693918
C	2.5669261	1.8835371	-1.6817131
C	2.321455	4.3622543	-0.6813913
O	1.2701801	-4.1565724	-1.005719
O	1.1051751	-1.9794136	-0.5433873
O	1.378257	0.5460222	1.6543517
O	2.4122239	-0.2919352	3.7459388
O	2.1023909	0.7657593	-1.8651646
O	3.5303631	2.3342503	-2.4059045
N	-0.2328783	-1.0094906	-2.5144495
N	-1.9208137	1.0121557	-1.9074553
N	-2.8856963	-0.7390721	0.4785665
N	-1.7810678	2.0645044	0.7898257
N	-0.5184958	-1.284956	2.0861912
N	0.811257	2.7424973	-0.1887201
Gd	0	0	0
